# Prediction of Extrusion Machine Stem Fatigue Life Using Structural and Fatigue Analysis

**DOI:** 10.3390/ma16083192

**Published:** 2023-04-18

**Authors:** Dong-Yul Kim, Ji-Wook Kim, Jin-Su Ha, A-Ra Jo, Sung-Yun Lee, Myeong-Sik Jeong, Dae-Cheol Ko, Jin-Seok Jang

**Affiliations:** 1 Gyeongbuk Research Institute of Vehicle Embedded Technology, Yeongcheon-si 38822, Republic of Korea; dykim@givet.re.kr; 2 Smart Manufacturing Technology R&D Group, Korea Institute of Industrial Technology, Daegu 42994, Republic of Korea; jwkim0@kitech.re.kr (J.-W.K.); jinsu302@kitech.re.kr (J.-S.H.); ooo7208@kitech.re.kr (A.-R.J.); yunskills@kitech.re.kr (S.-Y.L.); msjeong@kitech.re.kr (M.-S.J.); 3 Department of Nanomechatronics Engineering, Pusan National University, Pusan 46241, Republic of Korea

**Keywords:** extruder, structural strength analysis, fatigue analysis, fatigue life

## Abstract

In this study, the characteristics of the SKD61 material used for the stem of an extruder were analyzed through structural analysis, tensile testing, and fatigue testing. The extruder works by pushing a cylindrical billet into a die with a stem to reduce its cross-sectional area and increase its length, and it is currently used to extrude complex and diverse shapes of products in the field of plastic deformation processes. Finite element analysis was used to determine the maximum stress on the stem, which was found to be 1152 MPa, lower than the yield strength of 1325 MPa obtained from tensile testing. Fatigue testing was conducted using the stress–life (S–N) method, considering the characteristics of the stem, and statistical fatigue testing was employed to create an S–N curve. The predicted minimum fatigue life of the stem at room temperature was 424,998 cycles at the location with the highest stress, and the fatigue life decreased with increasing temperature. Overall, this study provides useful information for predicting the fatigue life of extruder stems and improving their durability.

## 1. Introduction

Recently, with the development of finite element analysis technology, as well as in the field of sintering processing such as forging, rolling, and extrusion, high-speed and high-load machine components are frequently used due to the advancement of industry [1].

As a result, metal materials experience fatigue failure due to continuous cyclic loads, which can lead to sudden failure. Therefore, there is an increasing demand for improved fatigue strength, fatigue life, and even the infinite life of metal materials [2].

The principle of extrusion is a sintering process that reduces the cross-sectional area and increases the length by inserting a cylindrical billet into a mold and pushing it out with a stem. Currently, complex and various shaped products are produced by extrusion [3].

There are several types of extrusion, including forward extrusion, backward extrusion, and hydrostatic extrusion. Forward extrusion is a process that compresses the material in the axial direction of the material and flows the material through a mold with a smaller forming section than the initial material diameter. Although there is almost no cracking in the material during forward extrusion, there are restrictions on the reduction rate due to the high average pressure [4].

Backward extrusion is a process that extrudes in the opposite direction of forward extrusion and has less material loss and extrusion pressure compared to forward extrusion [5].

Hydrostatic extrusion is a forming method that allows high-speed extrusion by being formed according to the shape of the mold when a material surrounded by a high-pressure fluid reaches the critical pressure. This improves the productivity and mechanical properties of the product [6].

In this study, an actual model of an extruder used in an industrial site that employs forward extrusion was selected for structural strength analysis, and the fatigue analysis using the stress–life method was conducted, and the fatigue life of the stem, which is a component subjected to load, was predicted.

The life of the mold is mainly affected by fatigue failure and wear, so it is important to design the mold considering the mold’s life and product dimensional accuracy. For extrusion molds, the evaluation of life can be largely divided into cases where the mold is destroyed due to the growth of fatigue cracks and cases where dimensional errors occur due to wear [7].

In general, fatigue analysis involves selecting an appropriate method between the stress–life (S–N) and strain–life (ε–N) approaches, considering the characteristics of the target material. The stress–life approach is suitable for long-life and constant amplitude load design of mechanical components or structures, as it involves simple analysis and evaluation of the required material constants. On the other hand, the strain–life approach is used for designs that involve low-cycle fatigue and consideration of plastic deformation [8].

## 2. Design and Structural Strength Analysis

### 2.1. Extruder Modeling

**Figure 1:**
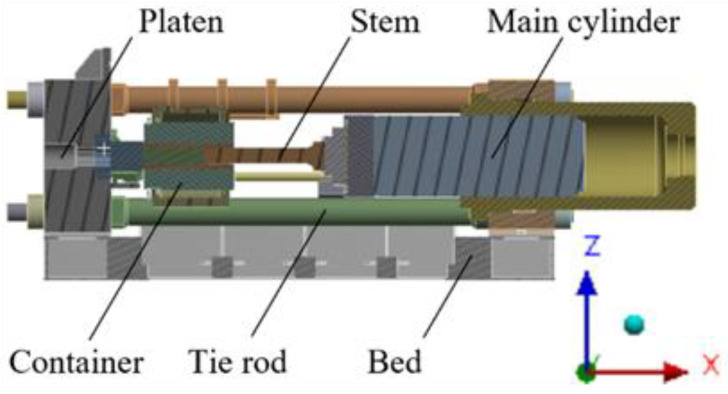
Components of an extruder.

The main components of the extruder include the main cylinder, stem, container, platen, tie rod, and bed, as shown in Figure 1. The extrusion process involves placing the material in the container, fixing it in place with connecting rods, and then pushing the stem through the main cylinder via the crosshead to shape the material through the die and discharge it through the platen. The overall size of the model is approximately 6.3 × 2.3 × 2.3 (m).

### 2.2. Finite Element Modeling

**Figure 2:**
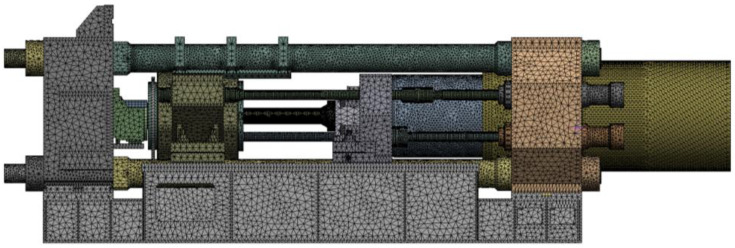
Finite element modeling of the extruder.

Structural strength analysis was performed using ANSYS 2021, a finite element software, and a finite element model was created as shown in Figure 2. The overall mesh size was set to 30 mm, and the size of the stem was set to 10 mm. The model consists of a total of 2,258,957 nodes and 1,344,621 solid elements. The stem is made of SKD61 material and material properties are summarized in Table 1.

Mesh size plays an important role in the analysis. Smaller mesh sizes give more accurate results but increase computation time, and larger mesh sizes reduce computation time but reduce accuracy. Therefore, choosing an appropriate mesh size is an important issue in numerical analysis because it balances computational time with an accuracy of results. The ratio of mesh elements is expressed as Jacobian Ratio, with an average value close to 1. The current model has an average ratio of 1.05, indicating a good size for modeling and meshing.

### 2.3. Boundary and Load Conditions

The lower part of the bed and the assembly parts that do not slip in the extrusion direction were constrained in all translational and rotational degrees of freedom. The parts where sliding occurs in the extrusion direction were constrained without considering friction.

**Figure 3:**
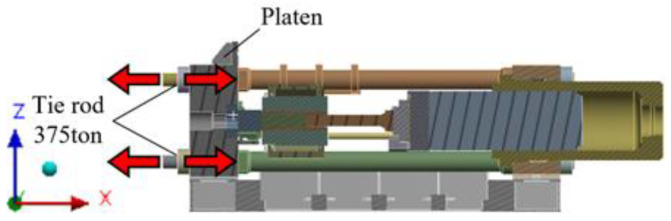
Tie rod load conditions.

**Figure 4:**
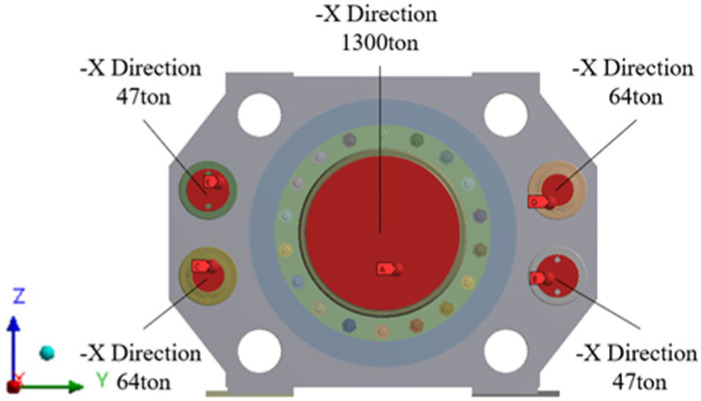
Cylinder load conditions.

Figure 3 shows the tie rod load conditions, and Figure 4 shows the cylinder load conditions. The load conditions applied were 375 tons each in different directions to the four tie rod nuts and horizontal column, 1300 tons to the main cylinder, and 64 tons and 47 tons to the container and side cylinders, respectively. Since the cylinder is subject to pressure, a load in the opposite direction was applied to the rear of the main cylinder and the inner surface of the cylinder housing, as shown in Figure 3 and Figure 4.

### 2.4. Results of Structural Strength Analysis

**Figure 5:**
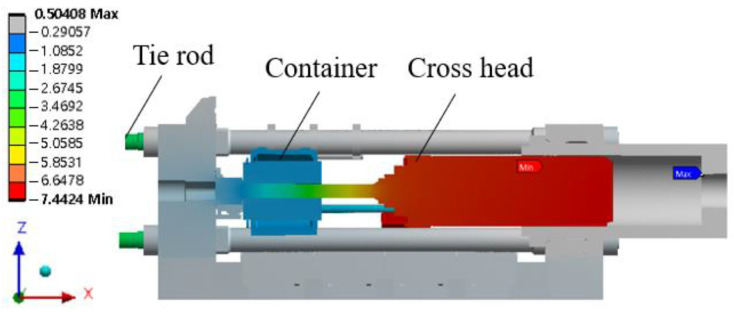
Total deformation of the X-axis.

The total deformation and stress distribution of the main components were analyzed when the extruder was subjected to loads. Figure 5 shows the total deformation along the X-axis. The container cylinder, which receives the load from the connecting rod, moved about 1 mm in the -X direction. The tie rod horizontal column, which is subjected to tensile stress, moved about 3 mm in the -X direction, and the crosshead body, which receives the load from the main cylinder, moved about 6 mm in the -X direction.

**Figure 6:**
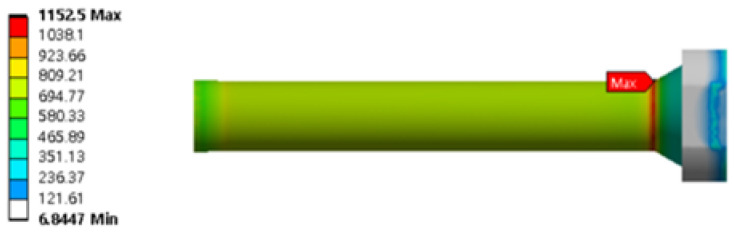
Von-mises stress of stem.

The maximum stress of the stem was 1152 MPa, which was below the yield strength value of SKD61, which is 1300 MPa. The safety factor was approximately 1.12, as shown in Figure 6.

The total deformation amount and stress distribution of major components were analyzed when the load was applied to the extruder.

Figure 5 shows the total deformation amount along the X-axis. The container cylinder, which receives the load of the connecting rod, moved about 1 mm in the -X direction. The tie rod horizontal column, which receives tensile load, moved about 3 mm in the -X direction, and the crosshead body, which receives the load of the main cylinder, moved about 6 mm in the -X direction.

The maximum stress of the stem was 1152 MPa, which is below the yield strength value of SKD61, 1300 MPa. The safety factor was about 1.12 and is shown in Figure 6.

**Figure 7:**
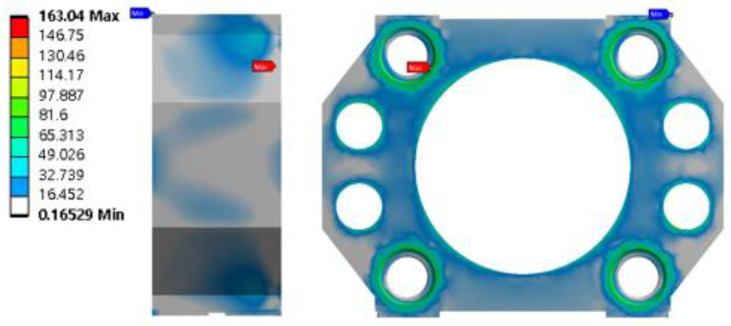
Von-mises stress of main cylinder housing.

The maximum stress of the housing was 163 MPa, which is below the yield strength value of SC46, 450 MPa. The safety factor was about 2.76 and is shown in Figure 7.

**Figure 8:**
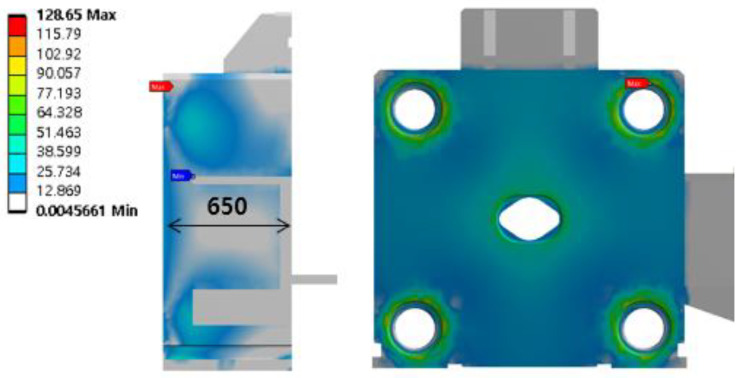
Von-mises stress of platen.

The maximum stress of the platen in Figure 8 was 128 MPa, which is below the yield strength value of SF55, 275 MPa, and it occurred at the tie rod nut. The safety factor was about 2.14, and the maximum stress and safety factor values are summarized in Table 2.

## 3. Tensile Test

### 3.1. Material and Test Specimen

**Figure 9:**
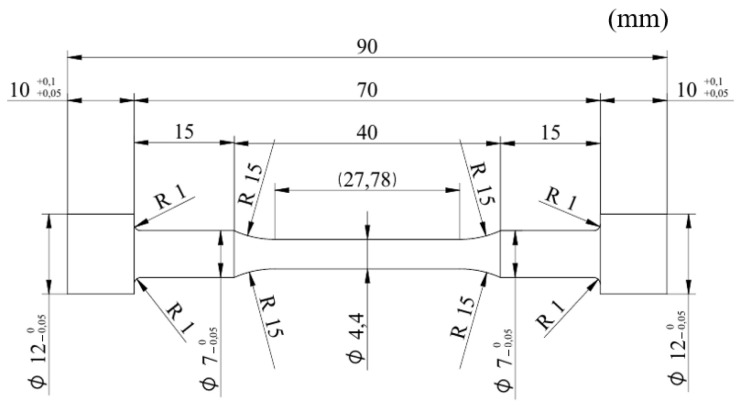
Configuration of the specimen.

The material used for the stem of this extruder is SKD61 steel, which is commonly used for hot extrusion and die casting molds as well as hot forging. The chemical composition is shown in Table 3 [9]. Tensile test specimens were fabricated as shown in Figure 9 to obtain the mechanical properties of the material.

### 3.2. Room Temperature Tensile Test

Tensile tests were conducted to obtain a load–displacement curve and material properties. First, the yield point, where the load decreases, is determined. Then, the load–displacement curve is converted to a nominal stress–nominal strain curve by dividing the load by the initial cross-sectional area and displacement by the initial gauge length from the start of the curve to the yield point. The elastic modulus is calculated by taking the slope of the linear region of the nominal stress–nominal strain curve, and the yield strength is determined using the 0.2% offset method [10].

**Figure 10:**
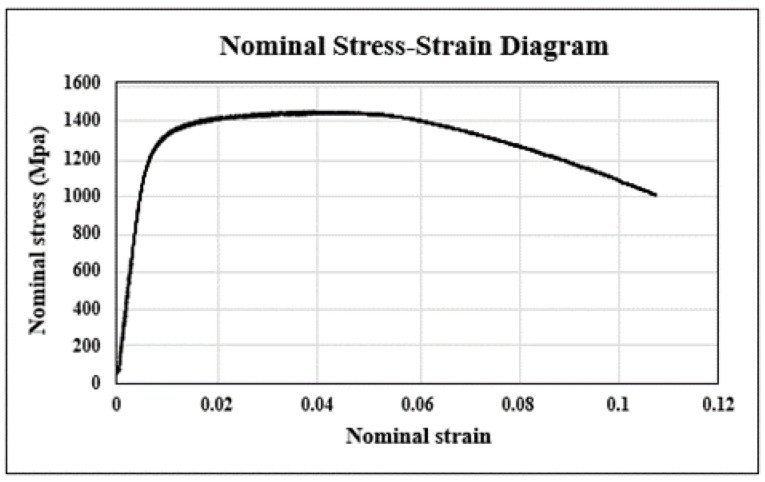
Nominal stress–strain graph of SKD61.

**Figure 11:**
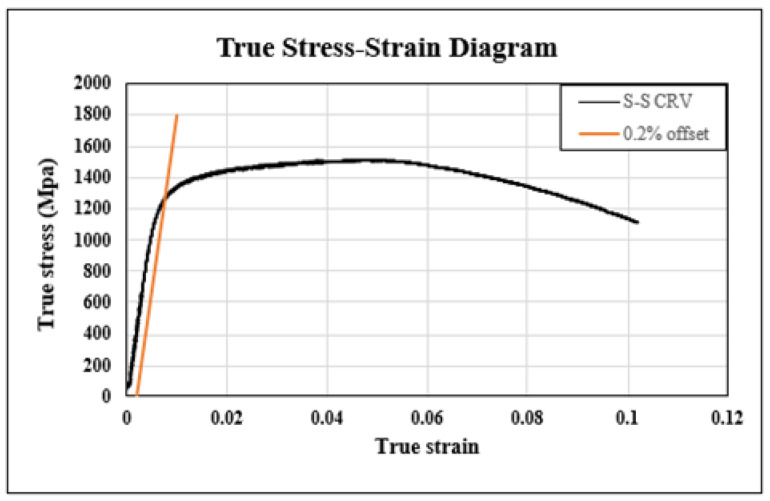
True stress–strain graph of SKD61.

The tensile test of SKD61 was conducted at room temperature, and the stress–strain curve is shown in Figure 10 for the nominal stress–nominal strain and Figure 11 for true stress–true strain. The elastic modulus was calculated to be 224,160 Mpa, and the yield strength was determined to be 1325 Mpa using the 0.2% offset method. The maximum tensile strength before fracture was approximately 1500 Mpa, and the results are summarized in Table 4. The difference occurs because the nominal stress is based on the cross-sectional area of the original specimen when calculating stress, and the true stress is based on the actual cross-sectional area, which continues to change during the tensile test.

### 3.3. High-Temperature Tensile Test

The high-temperature tensile test results refer to the research results of Yoh, E.G. [11]. SKD61 mold steel maintains the same mechanical properties at room and elevated temperatures, but a rapid deterioration of mechanical properties occurs when it reaches a certain temperature. At 300 °C and 500 °C, the reduction in mechanical properties with increasing temperature was relatively small, and the effect of the deformation rate was also negligible. However, a significant degradation of mechanical properties was observed at 700 °C and above.

## 4. Fatigue Test

### 4.1. Fatigue Failure and Fatigue Life

Fatigue is the phenomenon of material deformation that occurs even at stress levels lower than the tensile strength of the metal when it is subjected to repetitive loads. When a metal material is subjected to repetitive stress over a long period of time, fatigue progresses and eventually causes failure, which is known as fatigue failure. The number of cycles or time it takes for the material to fail under repetitive loads is called fatigue life [12,13,14,15,16].

### 4.2. S–N Curve

Fatigue tests measure the number of cycles required to cause the failure of the material under repeated loads and predict the fatigue life of the material through an S–N curve, which represents the relationship between the number of cycles to failure (N) and the alternating stress that causes failure when it is repeated [17,18].

### 4.3. Fatigue Test Method

The fatigue test was carried out in accordance with standards such as ASTM (American Society for Testing and Materials), JSME (Japan Society of Mechanical Engineers), and KS (Korea Standard), which describe the shape and number of specimens and the test methods for each type of test. In this study, the most widely used JSME S002 (1994) statistical fatigue test method was used, which includes 14 S–N test methods introduced by the staircase method [19,20].

**Figure 12:**
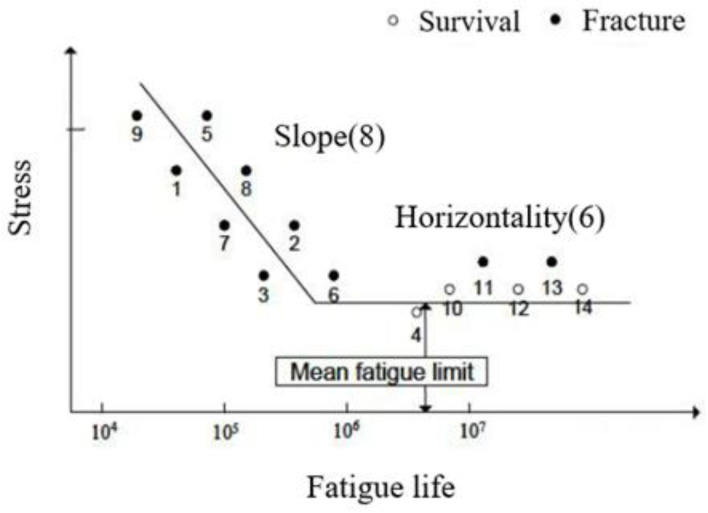
Statistical fatigue test of the S–N method.

Figure 12 shows the graph of the fatigue limit measured by the 14 S–N test methods, 8 of which are slope sections (finite life domain), and the remaining 6 are measured by the staircase method. Eight test specimens are used at four stress levels in the slope sections, with two specimens at each stress level. Six specimens are allocated to the horizontal section to obtain the median or average value of the fatigue limit, which is assumed to be 10^6^ [21,22].

### 4.4. Fatigue Test Results

**Figure 13:**
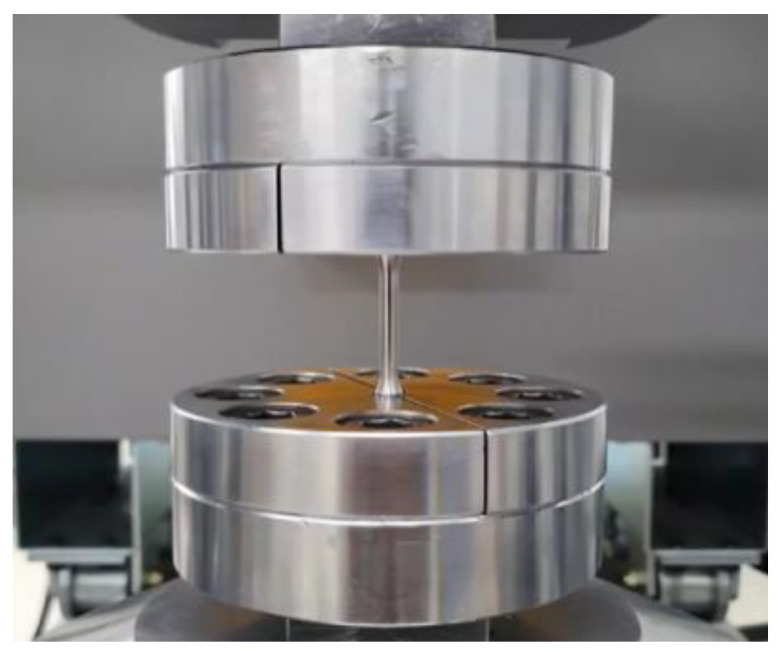
Fatigue test machine.

The specimens for the fatigue test were the same as the tensile specimens, and the fatigue load conditions were sequentially tested from 1100 Mpa, which is 75% of the tensile strength, and the stress ratio was fully reversed (R = −1) under the tension–compression condition. The test frequency was 10 Hz with reference to ASTM E466 [23], and the fatigue test picture is shown in Figure 13.

**Figure 14:**
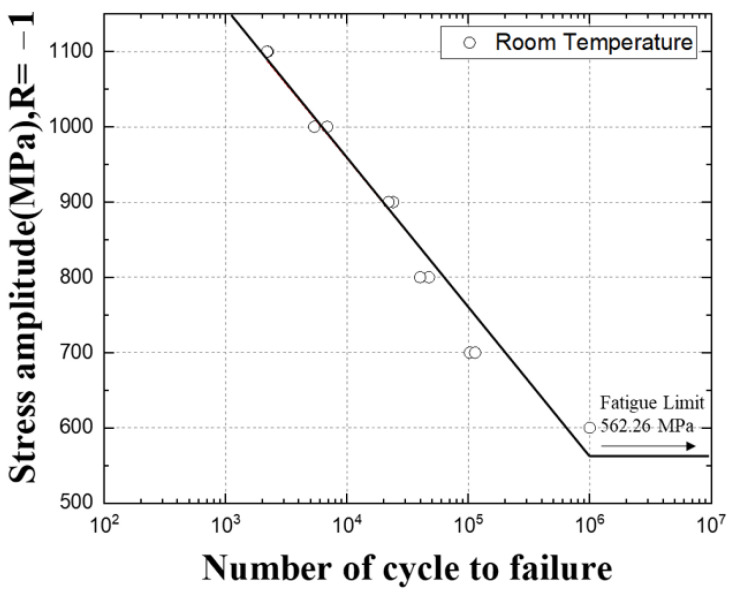
The S–N curve of SKD61.

The room temperature fatigue test results of SKD61 are summarized in Table 5. During the tensile test, the maximum tensile strength of the specimen was found to be 1500 Mpa, but it was confirmed that fracture occurred at 5375 to 6870 cycles during the fatigue test at 1000 Mpa, which is 70% of the tensile strength. Based on the fatigue test results, the S–N diagram was drawn in a log scale as shown in Figure 14, and the infinite lifespan was assumed to be 10^6^.

### 4.5. High-Temperature Fatigue Test Results

**Figure 15:**
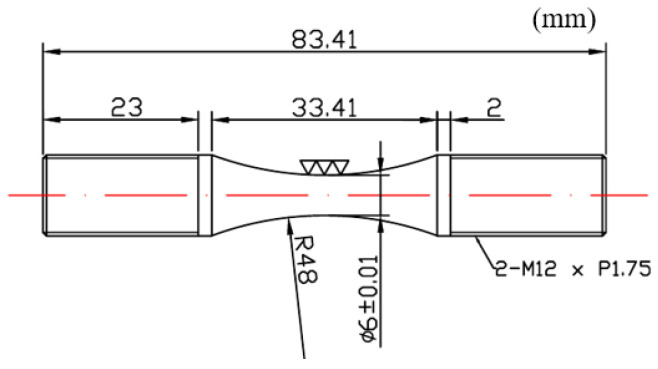
The fatigue test specimen at high temperature.

The specimens of the fatigue test are shown in Figure 15, and the temperature of the high-temperature chamber was tested in two types: 400 °C and 500 °C. The high-temperature fatigue load conditions at 400 °C and 500 °C were sequentially tested from 1000 MPa, which is 70% of the tensile strength.

The reason why the load condition was lower than that of the room temperature fatigue test was that the specimen fractured faster at a higher temperature than at room temperature. The stress ratio was conducted under the tension–compression condition. The test frequency was conducted at 10 Hz with reference to ASTM E466 at room temperature.

The results of the 400 °C high-temperature fatigue test are summarized in Table 6. During the tensile test, the maximum tensile strength of the specimen was 1500 MPa, but when the fatigue test was performed at 1000 MPa, which is 70% of the tensile strength, it was confirmed that the fracture occurred at a relatively lower cycle than the room temperature fatigue test, about 2908 to 3000 cycles.

The results of the 500 °C high-temperature fatigue test are summarized in Table 7. During the fatigue test at 1000 MPa, which is 70% of the tensile strength, it was confirmed that fracture occurred in fewer cycles than in the fatigue test at room temperature and 400 °C, about 154 to 465 cycles.

**Figure 16:**
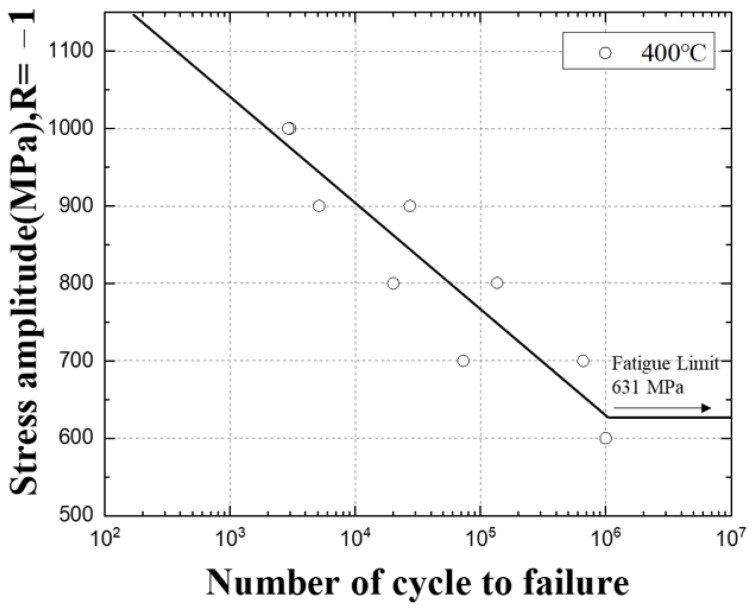
The S–N curve of SKD61 at 400 °C.

**Figure 17:**
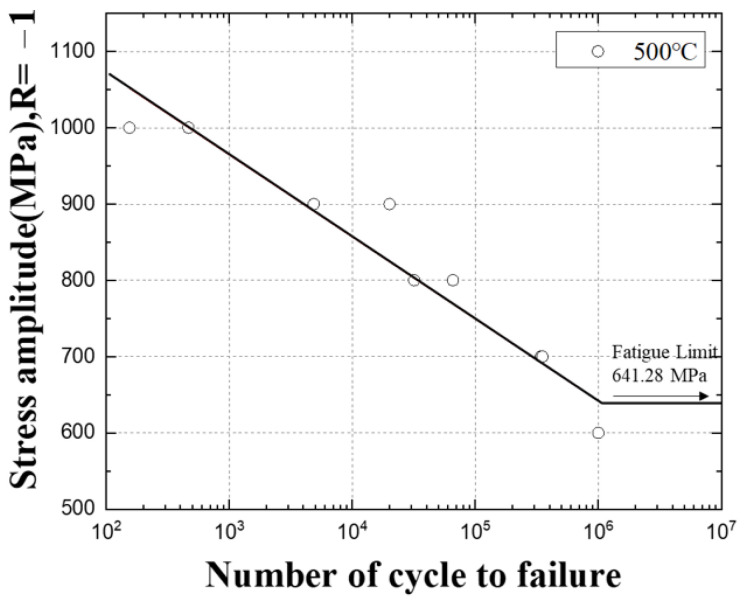
The S–N curve of SKD61 at 500 °C.

**Figure 18:**
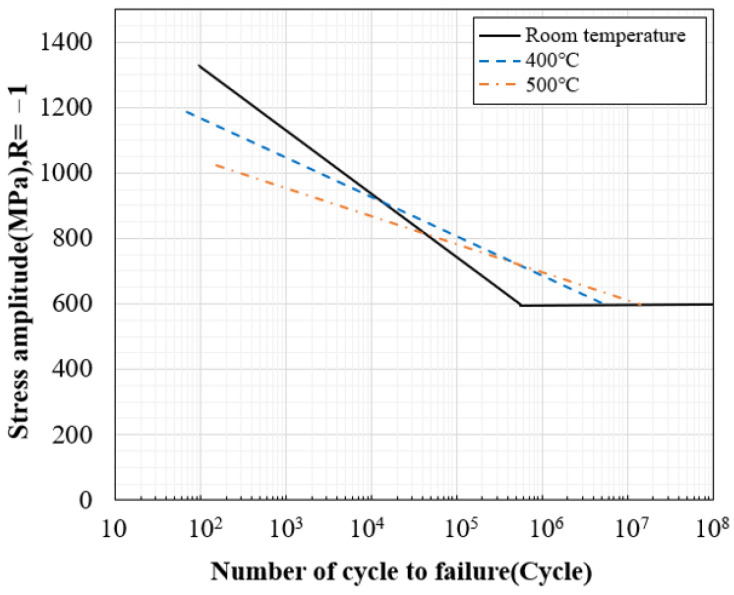
The S–N curve comparison graph of SKD61.

Based on the fatigue test results, the S–N diagrams in Figure 16 and Figure 17 were drawn in log scale, and the infinite lifespan was assumed to be 10^6^. In Figure 18, the fatigue test results at room temperature and high temperature were plotted as an S–N diagram.

When the temperature rises from room temperature to 400 °C and 500 °C, it can be seen that the slope of the S–N curve becomes gentle and the difference in the decrease gradually widens, and the number of cycles decreases at 1400~800 MPa stress. In addition, in the 800~600 MPa stress range, it can be seen that the number of cycles at room temperature is lower than at high temperature, and it seems to be an effect that changes from brittle fracture to ductile fracture as the temperature rises [11].

As the temperature increases, the strength of the material decreases, and at the same time, the rate of growth of defects or cracks within the material increases. This increases the risk of fatigue failure under high-temperature loading conditions. At high temperatures, the rate of shrinkage and expansion of the material increases, which reduces the stability of the material and causes the material to exhibit a more non-uniform stress state. When these factors combine, the reduction in fatigue life at high temperatures can be more pronounced. Therefore, materials such as SKD61 may have reduced fatigue life when used at high temperatures, as shown in the graph in Figure 18.

## 5. Finite Element Analysis of Fatigue Test Specimens

### 5.1. Finite Element Modeling of Fatigue Test Specimens

**Figure 19:**
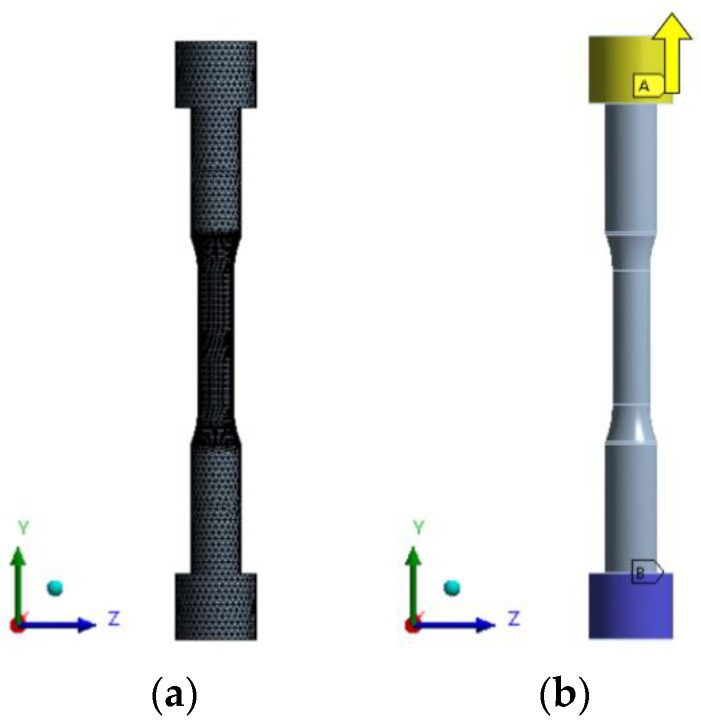
Finite element modeling and boundary conditions of tensile specimens: (**a**) Finite element modeling; (**b**) Boundary conditions.

For finite element analysis of the specimen, finite element modeling was performed as shown in Figure 19a, and the size of the grid was set to 1 mm. It was modeled with solid elements, and the total number of nodes was 118,650 and the number of elements was 80,850. For the property values, SKD61 was used, as shown in Table 1.

Boundary conditions are shown in Figure 19b. As in the tensile tester, the lower blue color constrains all the X, Y, Z translational and rotational degrees of freedom, and the upper yellow color gives the Y-direction displacement in the direction of the A arrow.

### 5.2. Fatigue Analysis Conditions of Test Specimen

The stress ratio used in the fatigue analysis was the same as that used in the fatigue test, which was conducted under tension–compression conditions. The fatigue strength factor (Kf) was input as 0.76.

Kf is a fatigue strength coefficient that represents the factors that affect fatigue failure, such as surface roughness, load, temperature, corrosion, and various defects. Generally, the results of fatigue tests for the same specimen can differ depending on the specimen’s condition. Kf represents the influencing factors for fatigue failure and is set to a value less than 1, which reduces the fatigue limit life.

‘Kf’ also affects the fatigue limit in the S–N diagram. If the numerical value of the influencing factor is input, the representative value of the factor can be calculated using Equation (1) [24,25]. There are various influencing factors, such as surface roughness (Ka), size factor (Kb), load factor (Kc), temperature, and corrosion. Surface roughness depends on the surface condition and tensile strength, and includes finishing operations such as grinding, machining, cold drawing, hot rolling, and forging. The size factor increases the probability of crack formation as the diameter of the structure increases because the stress gradient becomes smaller and the volume on which the maximum load is applied increases even with the same shape. The load factor depends on the type of loading (rotating, bending, axial) in the fatigue test.

(1) Kf=Ka×Kb×Kc×Kd×Ke

Ka = Surface factor,

Kb = Size factor,

Kc = Load factor,

Kd = Temperature factor,

Ke = Corrosion factor

### 5.3. Fatigue Analysis Results of the Fatigue Test Specimen

When a displacement of 0.214 mm in the Y direction of the tensile specimen was applied, a stress of about 1000 MPa was generated. Checking the fatigue test results of the tensile tester, when the fatigue test was performed at 1000 MPa, it was confirmed to be about 5375 to 6870 cycles and summarized in Table 8.

**Figure 20:**
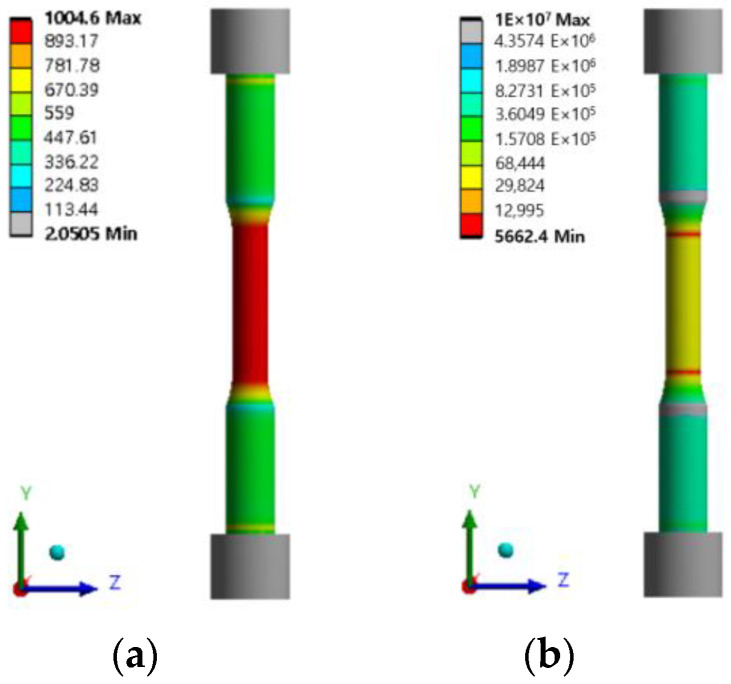
Results of stress and fatigue analysis of tensile specimens: (**a**) Von-mises stress of tensile specimens; (**b**) Fatigue analysis of tensile specimens.

When the fatigue analysis was performed based on the results of the finite element analysis of the tensile specimen, the minimum fatigue life was confirmed to be 5662 cycles, and the stress in Figure 20a and the fatigue life analysis results are shown in Figure 20b.

When comparing the finite element analysis results and the fatigue test results, it can be confirmed that they occur similarly within the error range.

## 6. Fatigue Analysis

### 6.1. Purpose of Fatigue Analysis

Fatigue refers to the failure that occurs when a material is subjected to fluctuating stress, even if the stress amplitude is less than the static strength of the material [26].

The stability of fatigue is one of the most important factors in ensuring the safety of machines and structural design, as most failures in machines or structures occur due to fatigue failure. In particular, machines that are in motion often experience a slow decrease in material strength over time, and it is often difficult to predict the time of failure. Fatigue failure typically occurs gradually without significant external signs, making it difficult to detect, and sudden failure can often result in serious accidents. Fatigue analysis is conducted to prevent unforeseen failure of materials when they are applied to practical machine components or structures, to predict the fatigue life and replacement timing of components, and ultimately to ensure safety and prevent physical and human damage. In order to conduct fatigue analysis, it is necessary to verify the basic material properties such as tensile strength, yield strength, and elastic modulus, as well as to understand the fatigue characteristics, based on the results of structural strength analysis, tensile testing, and fatigue testing.

### 6.2. Types of Fatigue Analysis

Fatigue analysis can be divided into stress–life (S–N) and strain–life (E–N) methods [27]. The stress–life method calculates fatigue life using calculated stress and stress–life curves and is used for evaluating the life of ductile materials that are subjected to long-cycle loading of more than 100,000 cycles. The strain–life method calculates fatigue life using a relationship between elastic–plastic strain and life, assuming that local plastic deformation affects fatigue life, and is used when subjected to low-cycle loading. In this study, the stress–life method (S–N method) was applied under long-cycle loading. The S–N method is a fatigue limit used for infinite life or safe stress design.

### 6.3. Effect of Mean Stress

In general, the fatigue limit is expressed as stress amplitude, and the stress–life method shows the alternating repetitive stress (S) against the number of repetitive loads (N) until failure as a graph, and the following relational expressions (2)–(6) represent the average alternating stress. It is used when explaining [28,29].

**Figure 21:**
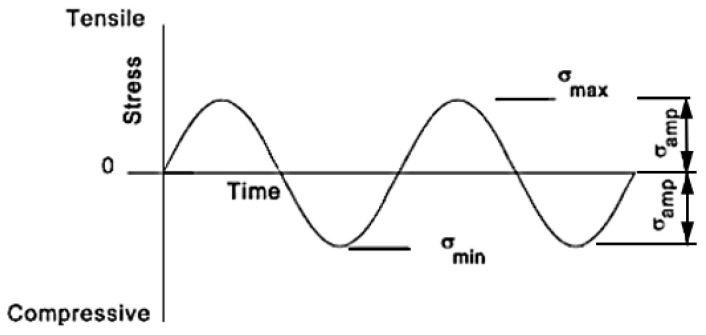
Fully reversed cyclic loads.

**Figure 22:**
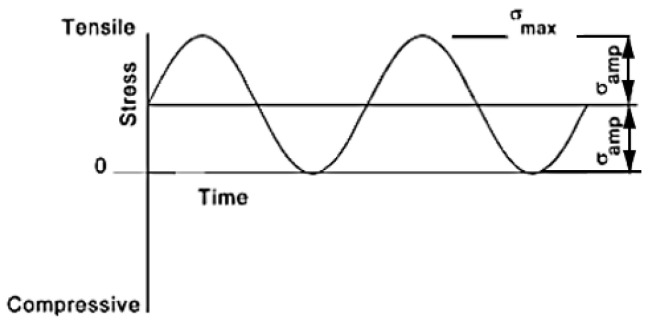
Tensile cyclic load.

The stress width in Equation (2) is the maximum stress minus the minimum stress, and the average stress in Equation (3) is the average value of the maximum and minimum stresses. The stress amplitude in Equation (4) is half the amplitude of fluctuating stress. The stress ratio in Equation (5) numerically indicates in which region the fluctuating stress amplitude is expressed as alternating load R = −1, tensile cyclic load R = 0, and compression cyclic load ∞. The amplitude ratio in Equation (6) is the value obtained by dividing the stress amplitude by the average stress, and the stress ratio and amplitude ratio according to general load conditions are shown in Table 9. In addition, the graphs of alternating load and tensile cyclic load are shown in Figure 21 and Figure 22 [30].

(2) $$\Delta \sigma =\sigma_{\max}-\sigma_{\min}$$

(3) $$\sigma_{m}=\frac{\sigma_{\max}+\sigma_{\min}}{2}$$

(4) $$\sigma_{a}=\frac{\sigma_{\max}-\sigma_{\min}}{2}$$

(5) $$R=\frac{\sigma_{\min}}{\sigma_{\max}}$$

(6) $$A=\frac{\sigma_{a}}{\sigma_{m}}$$

**Figure 23:**
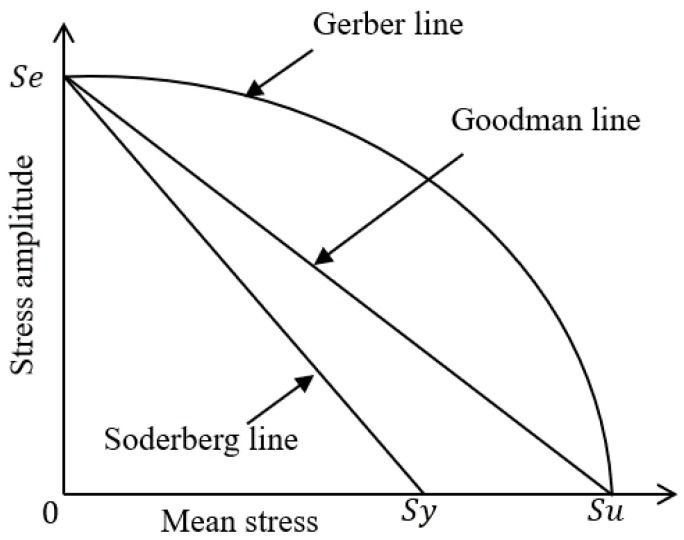
Comparison of mean stress equations.

The general characteristics of the effect of average stress are graphed in Figure 23, and representative relational expressions showing the effect of average stress are written in (7)–(9). Figure 23 shows the fatigue limit (Se), yield strength (Sy), and ultimate tensile strength (Su). Goodman’s theory of Equation (7) can obtain stable results for low ductility metals, and Soderberg’s theory of Equation (8) is more conservative than Goodman’s and is mainly used for brittle materials. The parabolic Gerber theory of Equation (9) is suitable for ductile materials in the state of tensile mean stress [31,32,33].

In this study, fatigue analysis was performed by applying Goodman’s theory to obtain results for ductile metal, and the amplitude ratio A = −1 was used because the load of the extruder stem is applied in the compression direction.

(7) $$\frac{\sigma_{a}}{S_{e}}+\frac{\sigma_{m}}{S_{u}}=1$$

(8) $$\frac{\sigma_{a}}{S_{e}}+\frac{\sigma_{m}}{S_{y}}=1$$

(9) $$\frac{\sigma_{a}}{S_{e}}+{\left(\frac{\sigma_{m}}{S_{u}}\right)}^{2}=1$$

### 6.4. Stress Concentration Factor

In most practical structures, loads are concentrated on a small area compared to the total area of the structure. To relieve stress concentrations, the design can be changed to distribute the load over the expected concentrated load points, or it can be designed with dimensions that gradually change angular edges [34,35].

### 6.5. Prediction of the Life of Extrusion Press Stem

**Figure 24:**
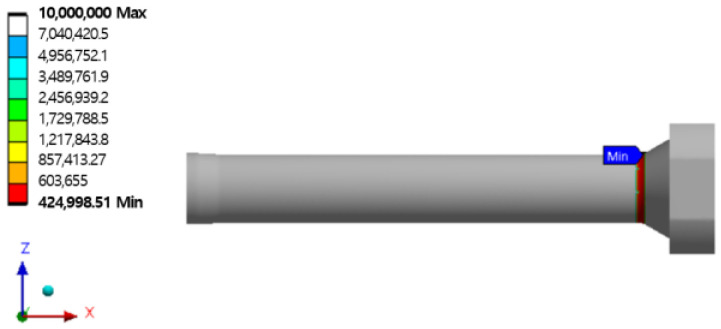
Results of the fatigue analysis of stem.

Fatigue analysis was performed to predict the life of the extrusion press stem using the stress–life (S–N) curve based on the results of structural strength analysis, tensile test, and fatigue test. The stress ratio was set to A = −1 and the Goodman method was used for mean stress correction, with a fatigue strength coefficient (Kf) input of 0.76 based on the finite element analysis results of the tensile specimen. The fatigue life analysis results are shown in Figure 24, with a minimum of 424,998 cycles at the location of the highest stress in the stem.

### 6.6. Heat Transfer Analysis and Results

In order to analyze the high-temperature stem life of the extruder, a structural heat transfer analysis was performed and the results were linked to a structural strength analysis. When heat is applied to the inner sleeve of the container surrounding the stem and the billet, heat transfer occurs within the equipment as it spreads to the surroundings. In the case of structural heat transfer analysis, the grid is generated and calculated only on the structure without considering the fluid area. This has the advantage of relatively reducing the calculation time compared to the fluid analysis, but since the fluid area is not calculated, it is difficult to accurately express the flow effect. However, assuming that the effect of convection on the fluid is small, the temperature change of the structure can be confirmed through structural heat transfer analysis.

**Figure 25:**
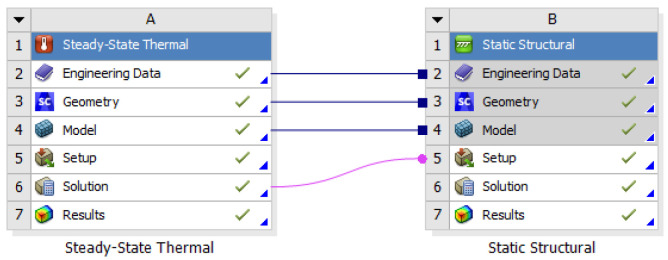
Heat transfer analysis process in ANSYS Mechanical.

**Figure 26:**
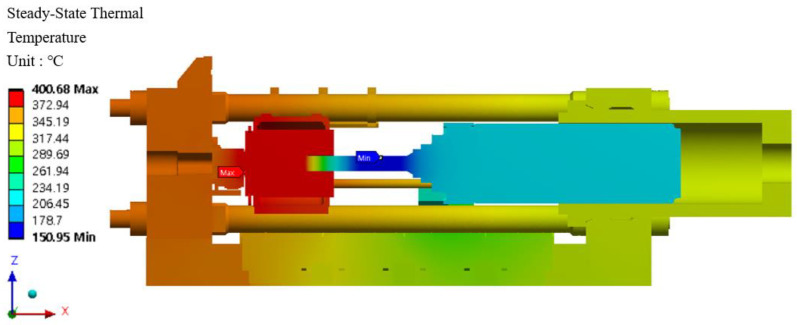
Results of the steady-state heat transfer analysis at 400 °C.

**Figure 27:**
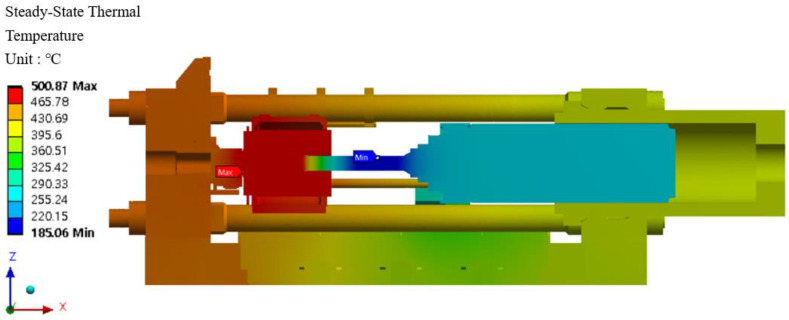
Results of the steady-state heat transfer analysis at 500 °C.

**Figure 28:**
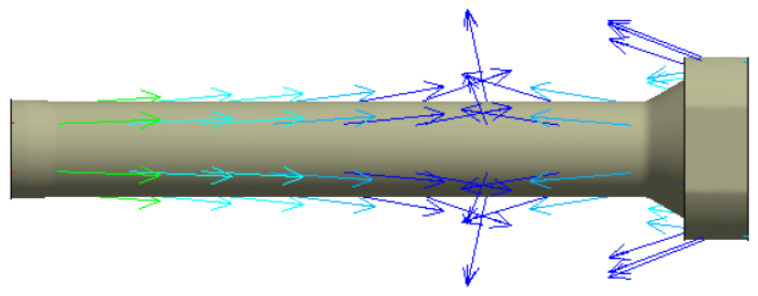
Steady-state heat transfer direction in the stem.

Heat transfer analysis is divided into steady-state heat transfer analysis and transient heat transfer analysis. The two-heat transfer analyses are determined by time dependence. A state in which the heat flow and temperature distribution are constant and do not change over time is called a steady-state heat transfer analysis. In order for this to happen, a transient heat transfer analysis must be performed. The purpose of heat transfer analysis is to apply heat to the billet during extrusion in the extruder so that temperature changes occur in the structure. Such temperature changes cause deformation or damage in severe cases. It is necessary to examine the mechanical properties such as the safety factor of the material. Since this extruder is a structure that extrudes at a constant temperature, a steady-state heat transfer analysis was performed. In addition, the distribution of the container temperature of the extruder during extrusion according to the extrusion process is 400–500 °C, and 400 °C and 500 °C were added to match the actual field equipment and finite element analysis conditions. The resultant data from the heat transfer analysis was linked to the structural strength analysis to evaluate the thermal strain and stress and is shown in Figure 25. The heat transfer analysis distribution is shown in Figure 26 and Figure 27 at 400 °C and 500 °C, respectively, and the direction of the heat transfer analysis result is indicated in Figure 28.

### 6.7. Extruder High-Temperature Stem Fatigue Life Prediction

Structural strength analysis was conducted based on the heat transfer analysis results of the extruder, and fatigue analysis was performed to predict the life of the extruder stem by applying the stress–life (S–N) diagram. A = −1 was used for the amplitude ratio, the Goodman curve was applied for the average stress correction, and 0.76 of the result of the finite element analysis of the tensile specimen was entered as the fatigue strength coefficient (Kf).

**Figure 29:**
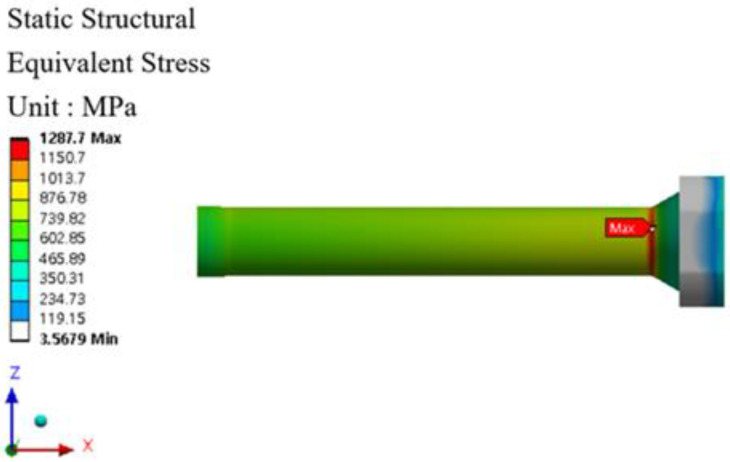
Von-mises stress of stem at 400 °C.

**Figure 30:**
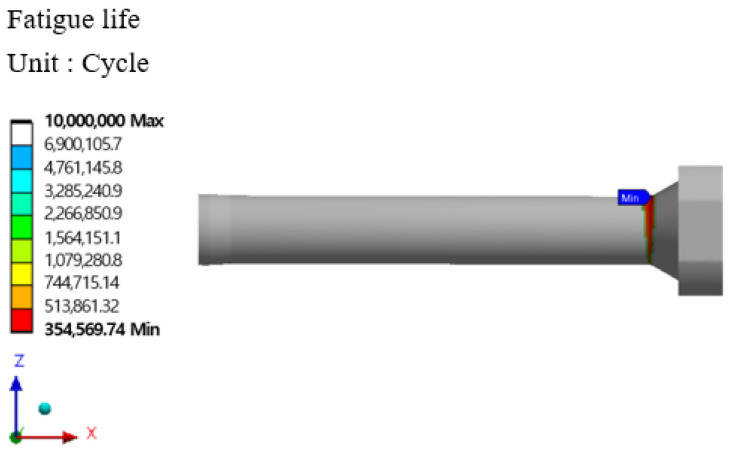
Results of the fatigue analysis of stem at 400 °C.

**Figure 31:**
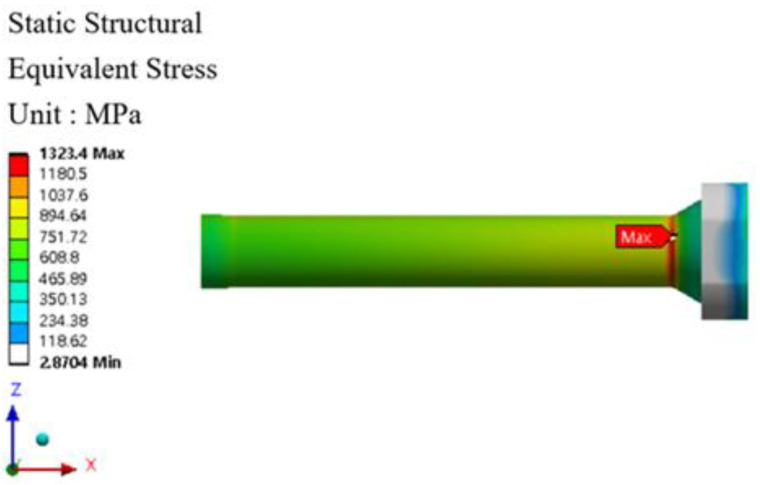
Von-mises stress of stem at 500 °C.

**Figure 32:**
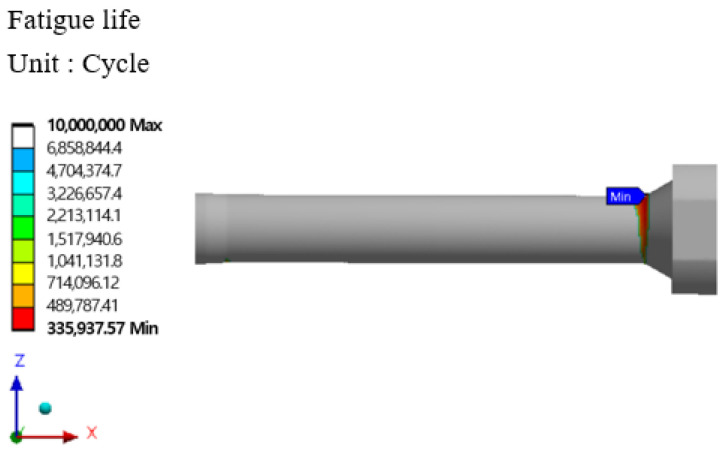
Results of the fatigue analysis of stem at 500 °C.

The stem stress and fatigue life analysis results at high temperatures are shown in Figure 29, Figure 30, Figure 31 and Figure 32. At the part of the stem with the highest stress, at least 354,569 cycles at 400 °C and at least 335,937 cycles at 500 °C were found. Table 10 summarizes the results of maximum stress and fatigue life.

The fatigue life of the stem was found to decrease as the temperature increased, consistent with the results of tensile tests. This indicates that temperature has an influence on the fatigue life of the stem. At low temperatures, the crystal structure of the metal is in a stable state, resulting in an increase in fatigue life. However, as the temperature increases, the crystal structure of the metal changes, and the fatigue life is expected to decrease.

## 7. Conclusions

In this study, structural analysis of the extruder and material analysis through tensile and fatigue tests were conducted, and fatigue life prediction of the stem was performed using finite element analysis.

(1) The main components of the extruder, such as the main cylinder, stem, and billet, were modeled and structural analysis was conducted to determine the maximum stress and safety factor. The maximum stress of the stem was 1152 MPa, which is below the yield strength value of SKD61 of 1300 MPa, and the safety factor was approximately 1.12.

(2) Tensile tests were conducted on SKD61 material used in the extruder stem to obtain the material properties and stress–strain curve. The results showed that the elastic modulus was 224 GPa, the yield strength was 1325 MPa, and the maximum tensile strength was 1500 MPa at room temperature.

(3) The fatigue test was conducted according to the JSME S002 (1994) statistical fatigue test method using a fatigue tester. During the fatigue test at 1100 MPa, which is 75% of the tensile strength, it was confirmed that fracture occurred at a relatively low cycle of about 2190 to 2227 cycles. The results of the high-temperature fatigue test at 400 °C showed about 2908~3000 cycles in the fatigue test at 1000 MPa and 154~465 cycles at 500 °C. As the temperature increased from room temperature to 400 °C and 500 °C, it was confirmed that the fracture occurred at a lower cycle.

(4) The fatigue test and the fatigue analysis were performed and compared, and the stress ratio in the fatigue analysis was conducted under the same tension and compression conditions as in the fatigue test. The fatigue strength factor (Kf) was applied at 0.76, and when checking the fatigue test results, when the fatigue test was performed at 1000 MPa, it was confirmed that it was about 5375 to 6870 cycles. When the fatigue analysis was performed on the tensile specimen with a load of 1000 MPa, the minimum fatigue life was found to be 5662 cycles, and it can be confirmed that it occurs similarly within the range of the fatigue test results.

(5) Fatigue analysis of the extruder stem was performed by applying the stress–life (S–N) curve prepared through the tensile test results and fatigue tests based on the structural strength analysis results. The stem fatigue analysis life at room temperature was at least 424,998 cycles at the highest stress area, at least 354,569 cycles at 400 °C, and at least 335,937 cycles at 500 °C.

Comprehensively analyzing the results, the maximum stress and safety factor were confirmed through the structural strength analysis of the stem, and a tensile test was conducted on the SKD61 material to identify the material’s characteristics and prepare a stress–strain curve. In addition, a fatigue test was conducted according to the JSME S002 (1994) statistical fatigue test method, and the fatigue test results at room temperature and high temperature were analyzed. Fatigue analysis of the extruder stem was performed by applying the stress–life (S–N) diagram prepared through the fatigue test, and the fatigue life tended to decrease as the temperature increased from room temperature to high temperature. It can be applied to the maintenance of the extruder stem to prevent safety accidents in advance, and additional experiments and analysis may be required for more accurate analysis.

## Figures and Tables

**Table 1:** Material properties.

Material	SKD61
Modulus of elasticity (GPa)	210
Poisson’s ratio	0.28
Density (kg/m^3^)	7810
Yield stress (MPa)	1325

**Table 2:** Results of the structural strength analysis.

Part	Maximum Von-Mises Stress (MPa)	Safety Factor
Stem	1152.5	1.12
Housing	163.0	2.76
Platen	128.6	2.14

**Table 3:** Chemical composition of the SKD61 steel.

Chemical Composition of SKD61 Steel					
C	Si	Mn	Cr	Mo	V
0.36	1.0	0.44	5.3	1.3	0.98

**Table 4:** Tensile test results of SKD61.

Materials	Modulus (Gpa)	Yield Strength (Mpa)	Ultimate Strength (Mpa)
SKD61	224	1325	1500

**Table 5:** The fatigue test result of SKD61.

Stress Amplitude (Mpa)	Fatigue Life, N (Cycle)	
1100	2227	2190
1000	6870	5375
900	24,000	21,961
800	47,287	39,990
700	102,510	113,680
600	10^6^↑	

**Table 6:** The fatigue test result of SKD61 at 400 °C.

Stress Amplitude (MPa)	Fatigue Life, N (Cycle)	
1000	3000	2908
900	5134	27,259
800	130,684	20,038
700	659,863	72,690
600	10^6^↑	

**Table 7:** The fatigue test result of SKD61 at 500 °C.

Stress Amplitude (MPa)	Fatigue Life, N (Cycle)	
1000	154	465
900	20,049	4849
800	65,812	31,792
700	340,240	351,138
600	10^6^↑	

**Table 8:** Comparison of fatigue test specimens and analysis results.

Stress Amplitude (MPa)	Fatigue Life of Fatigue Tester Specimen (Cycle)	Fatigue Life of Fatigue Analysis (Cycle)
1000	5375~6870	5662

**Table 9:** Stress ratio and amplitude ratio according to load condition.

Load Condition	Stress Ratio (R)	Amplitude Ratio (A)
Fully reversed	−1	∞
Zero to max	0	1
Zero to min	∞	−1

**Table 10:** Von-mises stress and fatigue life results according to the temperature change of the stem.

Temperature	Von-Mises Stress (MPa)	Fatigue Life (Cycle)
Room Temperature	1152.5	424,998
400 °C	1287.7	354,569
500 °C	1323.4	335,937

## Data Availability

The data presented in this study are available on request from the corresponding author and the first author.
